# Development and validation of a new rating scale for perimenopausal depression—the Meno-D

**DOI:** 10.1038/s41398-018-0172-0

**Published:** 2018-06-28

**Authors:** Jayashri Kulkarni, Emorfia Gavrilidis, Abdul-Rahman Hudaib, Caitlin Bleeker, Roisin Worsley, Caroline Gurvich

**Affiliations:** Monash Alfred Psychiatry Research Centre (MAPrc), Central Clinical School, Monash University and The Alfred Hospital, Melbourne, Victoria Australia

## Abstract

The menopause transition is a time when women experience an increased risk for new onset depression, as well as relapse of depression. While there are overlapping symptoms between major depression and depression during menopause, differences suggest ‘perimenopausal depression’ may be a unique subtype of depression associated with characteristic symptoms. There is currently no validated scale designed to measure perimenopausal depression. The aim of the current study was to develop and validate the ‘Meno-D’, a self-reporting or clinician rated questionnaire, designed to rate the severity of symptoms of perimenopausal depression. The development phase of the Meno-D involved literature review, clinical observation, and focus groups. A 12-item questionnaire was developed and clinically reviewed for face validity for content. The Meno-D was administered to women experiencing symptoms of perimenopausal depression as part of a larger baseline assessment battery. Validation involved confirmatory factor analysis (CFA). The development of the Meno-D resulted in 12 items. A total of 93 participants with perimenopausal depression were involved in the baseline assessments, 82 completed the Meno-D. Factor analysis identified five sub-scales of the Meno-D “somatic; cognitive; self; sleep; sexual” with high-internal consistency; discriminant validity and a good construct and convergent validity. The Meno-D provides a unique tool for clinicians and researchers to measure the presence of perimenopausal depression.

## Introduction

Women have approximately twice the risk of developing depression or anxiety disorders compared to men^1,2^. The menopause transition is a time when women are at an increased risk for new onset depression, as well as relapse for women with a history of depression^3–6^. While there are many overlapping symptoms between major depressive disorder and depression occurring during the menopause transition, there are also key differences that indicate ‘perimenopausal depression’ may be a unique subtype of depression^3^. The diagnosis and quantification of perimenopausal depression requires a new rating scale to reflect the unique subset of symptoms. This study presents the development and validation of a novel scale specifically designed to measure the severity of perimenopausal depression symptoms.

The perimenopausal period refers to the interval immediately preceding menopause, when women transition from a reproductive to a non-reproductive state, until menopause, when menses have ceased for a period of at least 12 months. The perimenopausal period typically begins for women during their mid-to late 40s with a number of physical and mental health changes which continue for ~4–5 years before menopause is reached^7^. The Stages of Reproductive Aging Workshop (STRAW) criteria provide the gold standard for characterizing reproductive aging through reproductive stages and menopause^8^. The STRAW recommends that the late reproductive stage is accompanied with subtle endocrine changes that transition into a perimenopausal period that includes an ‘early’ and ‘late’ transition phase as well as the ‘early’ stage of postmenopause^9^.

The perimenopausal period encompasses a range of endocrine changes that can be accompanied by varied biological and emotional symptoms. Menstrual cycle changes typically commence with irregularities in cycle length and progress to cessation of menses. Endocrine features include changes to FSH, estradiol, AMH, and ihibin-B; although, the STRAW recommendations are based on qualitative FSH criteria^9^. Vasomotor symptoms, comprising hot flushes and night sweats are the most common menopausal symptom^10^. Perimenopausal women are at an increased risk of developing depression and anxiety, as compared to pre or postmenopausal women, even after adjusting for variables such as personal history of depression, vasomotor symptoms, stressful life events and other demographic factors, such as age, race and socioeconomic status^5,11,12^. It has been estimated that 20% of perimenopausal women present to their primary healthcare physician with depressive symptoms^13^.

The spectrum of symptoms that are characteristic of perimenopausal depression include both physical and psychological symptoms. Muscle pain, weight gain, low energy levels, decreased self-esteem, feelings of isolation, cognitive impairment, and decreased libido have all been described in women with perimenopausal depression^3,14,15^. Although these symptoms overlap with major depressive symptoms endured by women not experiencing menopause, some key characteristics appear to differentiate perimenopausal depression. Perimenopausal depression is associated with a milder mood presentation when compared to depression experienced by women during childbearing years^16^. Major depression is often characterized by sadness; whereas, the mood symptom profile associated with perimenopausal depression comprises anger, irritability, and paranoia which may manifest as verbal outbursts often over minor stressors, and out of character of the women experiencing these symptoms^17^. Worsley et al.^17^ described their observations of perimenopausal women who experience episodes of mood changes as an ‘on–off’ phenomenon that may last for min to hours and then spontaneously resolve. In comparison to childbearing aged depressed women, perimenopausal depression is associated with increased fatigue and decreased energy levels that are independent of sleep disturbance^3,16–18^. Hence, while further research is needed to categorically differentiate perimenopausal depression from other depression subtypes there does appear to be a unique symptom profile associated with perimenopausal depression.

The etiology of perimenopausal depression fits well within a biopsychosocial model. Significant hormonal changes occurring in the hypothalamic–pituitary–gonadal axis have direct effects on brain regions and neurotransmitter systems involved in the modulation of mood^19^. For example, there is now a substantial evidence base supporting the influence estrogens and progesterone have on numerous CNS processes (such as reducing inflammation, increasing neurogenesis, and neuronal regeneration), as well as their capacity to modulate dopaminergic and serotonergic transmission^20^. Gonadal hormones also interact with the other major endocrine axis, the hypothalamic–pituitary–adrenal axis, with evidence indicating hypothalamic–pituitary–adrenal axis dysregulation may contribute to increased depression risk or exacerbated sensitivity to stressful situations^21^. A further biological influence on depression risk is age at onset of menopause, as well as the duration of the reproductive years. Older age at menopause, and a longer reproductive period have both been associated with a reduced risk of menopausal depression^22^.

These biological influences and predispositions are likely to interact with several psychosocial factors that coincide with menopausal years such as perception of aging and childbearing status, life habits, and stressful family/life roles. The societal and individual perspective on age, such as the tendency to value youth more than the elderly, can impact the likelihood of depression during menopause^3^. Communities that place more value on elders have lower rates of depressive symptoms during perimenopause. Smoking and limited physical activity may also leave a woman susceptible to these symptoms^23^. Additionally, the stress of caring for children or elderly parents, or disharmonious family relationships have been linked to higher rates of depressive symptoms during perimenopause^3,24^.

Collectively, research suggests that perimenopausal depression is a subtype of depression with a unique etiology and specific symptom characteristics. In line with this, there are several studies indicating that women experiencing perimenopausal depression may respond differently to antidepressant medications as compared to women experiencing depression outside of the menopause transition^25^. Future research is needed to determine optimal clinical management of perimenopausal depression, considering risk-to-benefit ratios of hormonal compared with antidepressant therapies, as well as psychosocial interventions.

Over the past decade, interest in the relationship between depression and perimenopause has increased. While a number of validated depression rating scales exist, there is no validated scale specifically designed to measure or monitor the symptom profile associated with perimenopausal depression. For example, depression scales, such as the Beck Depression Inventory II^26^ or Montgomery and Asberg Depression Rating Scale^27^ (both often used in this population) do not have questions specifically targeting paranoid thinking, memory problems or the experience (rather than impact of) somatic symptoms that are specific and critical to the depression experienced in menopause. The Menopause-Specific Quality of Life (MENQOL)^28^ questionnaire captures some aspects of depression, anxiety, poor sleep, and poor memory, but does not specifically rate concentration problems, self-esteem or social withdrawal. Hence, the aim of the current study was to develop and validate a questionnaire, called the “Meno-D” as shown in Table 1, designed to capture and rate the severity of the characteristics symptoms of perimenopausal depression.

**Table 1:** MENO-D

A: Low energy		
Over the last 2 weeks: How has your energy been? -Did you feel more tired after activity than normal? -Did your activity decrease because you were tired? -Did you feel tired most of the time despite decreasing your activity? -Did you continually feel tired so that even small tasks like brushing your hair felt draining?	0	No change in energy, feel active all day
	1	More tired after activity than previously
	2	Decreased activity because of tiredness
	3	Feel tired most of the time despite resting, decreased activity
	4	Continually feeling exhausted, even small tasks such as brushing hair feel draining. “Bone weary, mind weary”

B: Paranoid thinking		
Over the last 2 weeks: -Have you been feeling guilty? -Have you been worried that others think badly of you? -Have you been suspicious that others think badly of you? -Have you been convinced that others have a low opinion of you or are trying to replace you?	0	No Paranoid thinking
	1	Increasingly worried that others think badly of you
	2	Suspicious that people at work or home think badly of you
	3	Convinced that others have a low opinion of you and are trying to replace you
	4	Convinced that others are actively planning to hurt you in many ways

C: Irritability		
During the last 2 weeks: -Have you felt more irritable than usual? -Have you snapped at anyone or been short with anyone over small incidents? -Have you felt real rage and had major outbursts about minor incidents?	0	No irritability
	1	Mild irritability
	2	Increased irritable response to minor incidents
	3	Anger expressed by “snapping”, verbal outbursts over minor incidents
	4	Rage, major verbal outbursts over minor incidents

D: Self-esteem		
Over the last 2 weeks have you: -Felt worse about yourself than usual? -Felt really bad about yourself? -Felt worthless and made negative comments about yourself? -Believed that the world would be better off without you? -Harmed yourself in any way? -Planned suicide? -Attempted suicide?	0	Good self-esteem or no change in self-esteem
	1	Slight decrease in self-esteem
	2	Poor self–esteem with no reality base
	3	Very poor self-esteem in all life domains, with marked self-denigratory comments
	4	No self-worth at all to the point of believing that the world would be better off without you. (NB—this rating must then lead to further questions about suicide planning, actions and deliberate self-harm)

E: Isolation		
Over the last 2 weeks have you: -Been socializing as normal? -Had less of an interest in socializing? -Become socially withdrawn? -Felt isolated, even when with others?	0	Socialize normally
	1	Decreased socializing
	2	Disinterested in socializing
	3	Social and occupational withdrawal
	4	Feeling isolated, “in a bubble” even when with others

F: Anxiety		
Over the past 2 weeks have you: -Felt especially anxious or nervous when in public? -Felt highly anxious when completing new tasks? -Felt highly anxious when completing tasks that are routine or familiar to you? -Had panic attacks and felt extremely anxious when doing normal everyday things?	0	No new anxiety
	1	Increased anxiety when performing in public
	2	Highly anxious when doing new tasks
	3	Heightened anxiety when doing routine and familiar tasks
	4	Panic attacks, highly anxious when doing ordinary and familiar tasks

G: Somatic symptoms		
Over the last 2 weeks have you: -Had any physical complaints? -Had increased physical pain with little exertion? -Experienced frequent headaches or joint and muscle pain that limited your activity? -Experienced severe and debilitating aches and pains that prevented you from engaging in activity?	0	No physical symptoms
	1	Increased muscle aches, joint pains on exercise
	2	Increased leg, back and joint pains with little exertion
	3	Frequent headaches, muscle and joint pains limiting activity
	4	Severe aches and pains requiring pain relief and preventing activity

H: Sleep disturbance		
Over the last 2 weeks: -How has your sleep been? -Has your sleep been broken briefly but you could get back to sleep easily? -Has your sleep been broken several times each night and you found it hard to get back to sleep? -Have you been waking up more than 2 or 3 times per night due to hot flushes, sweating? -Have you on most nights been sleeping for only 2 h or less due to sweating, hot flushes, and night chill	0	No sleep problems
	1	Sleep broken by brief waking once or twice per night, but easily return to sleep
	2	Sleep broken by waking several times per night, but easily return to sleep
	3	Waking up three or more times per night due to hot flushes and sweating, plus difficulty returning to sleep
	4	Sleeping two or less hours per night consistently. Sweating, hot flushes, feeling hot then cold, interrupting sleep all night

I: Weight		
Over the past 2 weeks: -Has your weight changed at all? -How much? -Have you gained a moderate amount of weight despite no change in diet or exercise? -Have you continued to gain weight despite engaging in strict dieting or increased exercise? -Have you had a major weight gain of 6 kg or more?	0	No change in weight
	1	Mild weight gain (1–2 kg)
	2	Moderate weight gain despite no change in diet or exercise (3–6 kg)
	3	Continuing weight gain and abdominal fat deposition, despite dietary restriction and increasing exercise
	4	Major weight gain (>6 kg) with abdominal, breast, hip, and thigh fat deposition

J: Sexual interest		
Over the past 2 weeks: -Have you had any change in libido? -Have you had decreased libido? -Has your libido diminished significantly? -Have you had discomfort with sexual activity in addition to a decreased libido? -Have you lost all interest in sexual activity?	0	No change in libido
	1	Mild decrease in libido
	2	Diminished libido
	3	Decreased libido and discomfort with sexual activity
	4	Loss of interest in all sexual activity

K: Memory		
Over the last 2 weeks; -Have you noticed any change in memory? -Did you have mild problems remembering simple things like names and numbers? -Did you need to make lists in order to function at work or at home? -Did memory problems lead to dysfunction or impairment in any way?	0	No change in memory
	1	Mild problems remembering names and numbers
	2	Need to make lists to function at work or home
	3	Impaired memory leading to dysfunction
	4	Severe loss of memory leading to inability to function

L: Concentration		
Over the past 2 weeks; -Have you had any problems concentrating? -Did you have difficulty reading or holding a conversation? -How severe were these problems? -Were you unable to focus on any task for a suitable period of time?	0	No change in concentration
	1	Mild problems with concentrating on reading
	2	Mild problem with concentration on reading and watching TV/films
	3	Marked problems concentrating on reading and watching TV/films
	4	Unable to focus on any tasks

## Method

### Questionnaire development

The psychological symptoms of menopause were identified through literature review, clinical observation, and experience plus the use of focus groups, which included perimenopausal women mental health clinicians and physicians. Careful attention was paid to symptoms experienced by women between 43 and 54 years, in both physical and mental health domains. Themes of commonly repeated symptoms were identified and developed into the questionnaire. Twelve symptom areas were identified, which are related to energy, paranoia, irritability, self-esteem, isolation, anxiety, somatic symptoms, sleep, weight, sexual interest, memory, and concentration. Each of the 12 symptoms, are rated on a scale from 0 to 4. The total score can range from 0 to 48.

Face validity of the questionnaire content was obtained by review and discussion with 6 psychiatrists, 10 mental health nurses and 2 endocrinologists. The Meno-D questionnaire was designed to be used either as a self-reporting tool, or as a questionnaire to be delivered by a clinician during an interview with the woman.

### Meno-D data validation studies

The Meno-D was administered as part of a baseline assessment for two separate studies (see inclusion/exclusion criteria in Supplementary material). The data were pooled and analyzed. Both studies were recruiting women experiencing symptoms of perimenopausal depression and are aged between 45 and 65 years. The first study was a randomized controlled trial investigating a novel treatment for perimenopausal depression and only baseline data for 39 perimenopausal women was gathered and used for the analysis (Clinical trials registration number ClinicalTrials.gov: *NCT01470092)*. The second study is looking at factors that increase the risk of anxiety in perimenopausal women and 54 patients and their data were used in this study. This provided a total of 93 patients. Both studies involved a range of clinical interviews that were completed at the initial/baseline assessment catered for individual study needs. The studies were approved by The Alfred Hospital Human Research Ethics Committee, Melbourne Australia. All participants provided written informed consent (according to the guidelines of the Australian National Health and Medical Research Council).

Of relevance to the current study, the MINI^29^ was used to confirm diagnosis (e.g., MDD) or confirm that participants did not meet other DSM IV diagnostic criteria (No Diagnosis category) and data are presented in Table 2 to demonstrate the diagnostic spread of the current sample. The STRAW^30^ was used for menopause staging and basic demographic information was incorporated into the analyses.

**Table 2:** Demographic information

	Reproductive late	Menopausal transition early	Menopausal transition	Postmenopause early	Postmenopause late	Total *n* = 83
Total *n*	*n* = 3	*n* = 36	Late *n* = 27	*n* = 7	*n* = 10	
*Relationship status*						
Single	1	7	3	0	2	13
Married	2	18	19	4	4	47
De Facto	0	3	3	2	2	10
*Divorced/separated*	*0*	*6*	*1*	*1*	*1*	*9*
Widowed	0	1	1	0	0	2
Never Married	0	1	0	0	0	1
Missing	—	—	—	—	1	1
*Ethnicity*						
Caucasian	3	31	21	7	9	71
East/South East Asian	0	3	3	0	0	6
African	0	0	1	0	0	1
Other	0	2	2	0	1	5
*Diagnosis*						
MDD	2	13	7	7	10	39
Dysthymia	0	0	1	0	0	1
Anxiety disorder	0	6	4	0	0	10
Other	1	1	2	0	0	4
None	0	15	13	0	0	28
Missing	—	1	—	—	—	1
*Age (years)*						*83*
Mean	47.00	47.83	51.04	53.71	57.80	
Std. deviation	2.646	2.772	3.044	2.812	3.994	
Std. error	1.528	.462	.586	1.063	1.263	
*BMI*						*46*
Mean	—	25.598	25.218	—	—	
Std. deviation	—	5.159	4.04*5*	—	—	
Std. error	—	1.032	.883	—	—	
*Missing data*	*3*	*11*	*6*	*7*	*10*	*37*

### Data analysis

The factor model was explored with confirmatory factor analysis (CFA) conducted with AMOS (version 22) using the Maximum likelihood estimation. The data’s covariance fit for the tested factor models was examined. A five factor model was deemed to be conceptually compatible with the theory framework that stem from the literature review of perimenopause depression syndromes and the vast clinical expertize of the focus group (psychiatrists, clinicians, and social workers) who were involved with the scale development. In this study, the five factor model was compared to a default single-factor model.

The two models were compared using the following criterion-based and goodness of fit indices: model *χ*^2^ statistic *χ*^2^, *χ*^2^ statistic *χ*^2^ divided by the degree of freedom (*χ*^2^/df), Akaike’s information criterion (AIC), comparative fit index (CFI), Tucker-Lewis Index (TLI) and root mean square error of approximation (RMSEA). For the AIC, and *χ*^2^/df, lower values indicate better fit. For the CFI, TLI, values >0.90 represent a good fitting model. For RMSEA, values <0.1 are consistent with acceptable model fitness^31^.

Furthermore, convergent validity was assessed by the average variance extracted (AVE) with values above .50 demonstrating a convincing and compelling evidence of convergent validity^32^. Internal consistency was evaluated through composite reliability (CR) with values above .70 indicating good internal consistency^33^. Discriminant validity was assessed by the heterotrait-monotrait ratio (HTMT) of the correlations as proposed by Henseler et al.^34^. The discriminant validity (by the HTMT method) compared the model HTMT ratio to the threshold of .85^35^. This was performed with SmartPLS software.

## Results

### Participants

The final sample included 93 patients, of which 82 completed the Meno-D. The mean age of the respondents was 50.54 (SD = 4.446), mostly married (57%) and the majority was Caucasian (87%). The characteristics of the respondents are shown in Table 2.

The results for the CFA for the two models, are shown in Table 3. In the one factor model, all item-factor loadings (standardized regression weights) were significant (*p* < 0.05). However, items 7 (Somatic symptoms), 9 (weight changes), and 10 (sexual interest) showed poor factor loadings (<0.40). Furthermore, the fit indices indicated unacceptable factor solution. See Table 3.

**Table 3:** Confirmatory factor analysis results

Model	df	*χ* ^2^	*χ*^2^/df	AIC	CFI	TLI	RMSEA (90% CI)
One factor	54	110.72*	2.05	182.72	0.84	0.77	0.11 (.08–.14)
Five factor	44	50.73	1.15	142.47	0.98	0.96	0.04 (.0–.08)

*AIC* Akaike’s information criterion, *CFI* comparative fit index, *TLI* Tucker-Lewis Index, *RMSEA* root mean square error of approximation

*p < 0.05

**Fig. 1:**
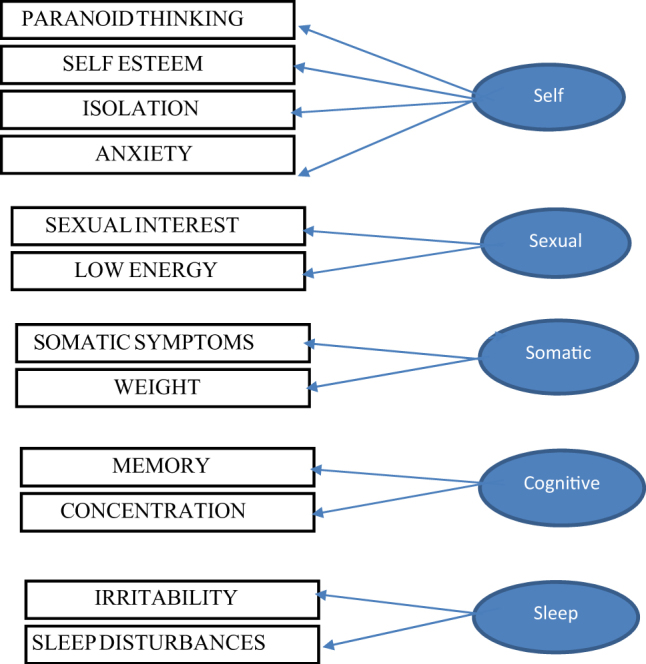
Schematic representation of the 5-factor model

The five factor model item-factor loadings were significant (*p* < 0.001). Items 2 (Paranoid thinking), Item 4 (Self-esteem), Item 5 (Isolation) and Item 6 (Anxiety) loaded highly (>0.70) on a common latent construct named “Self”. Items 7 (Weight changes) and Item 9 (Somatic symptoms) loaded highly on another construct named “Somatic” factor. However, Item 10 (Sexual interest) and Item 1 (low energy) loaded on a separate factor named “Sexual” factor. Items 11 and 12 (representing memory and concentration, respectively) both loaded highly on a factor named “Cognition”. Item 8 (sleep disturbances) and Item 3 (irritability) loaded on a factor named “Sleep” (Fig. 1). There were no cross loadings of items on factors. The model fit indices were acceptable and superior to the one factor model. Results are shown in Table 3.

### Validity and reliability

As Table 4 shows, the five sub-scales of the Meno-D exhibit high-internal consistency with values of composite reliability above the cutoff point of .70. Results from the HTMT ratio of correlations indicated discriminant validity. The AVE values were above the threshold of .5 suggesting a good construct and convergent validity^35^.

**Table 4:** Reliability and validity

	Discriminant validity-HTMT					AVE	CR
	Self	Sexual	Somatic	Cognition	Sleep		
Self						0.72	0.91
Sexual	0.69					0.71	0.83
Somatic	0.42	0.41				0.76	0.86
Cognition	0.75	0.78	0.30			0.79	0.88
Sleep	0.68	0.47	0.64	0.76		0.69	0.82

*AVE* Average variance extracted, *CR* composite reliability

## Discussion

Recent literature suggests that perimenopausal depression is a unique subtype of depression, with characteristic symptoms, etiology, and course that are distinct from other depression subtypes. The primary aim of the current study was to develop and validate a 12-item questionnaire (either self-administered or clinician rated) to measure and rate the severity for perimenopausal depression, the Meno-D. Factor analysis identified five sub-scales of the Meno-D “self; somatic; cognitive; sleep; sexual” with high-internal consistency; discriminant validity and a good construct and convergent validity.

Factor analyses indicated that the Meno-D is composed of five factors, each with good reliability. The first factor represents ‘self’ and is comprising the following items: self-esteem, isolation, paranoid thinking, and anxiety and allows assessment of whether the patient is experiencing paranoid thinking such as guilty feelings or suspicious thoughts, decreased self-esteem (poorer self-esteem), less interest in socializing and feelings of anxiety. The second factor, ‘sexual’ refers to changes in libido and sexual activity as well as assessing whether the patient has experienced a decrease in energy. The third factor, ‘somatic’ reflects somatic symptoms, any physical pain and changes in weight. The ‘cognitive’ factor captures any subjective changes in memory and concentration and the final factor ‘sleep’ can monitor any increases in irritability and/or sleep disturbances.

Previous studies have reported that women experiencing perimenopausal depression complain about physical symptoms more than cognitive ones, which are not typically included in previous scales assessing for major depressive disorder^28^. This may explain why perimenopausal depression is often overlooked or left undiagnosed. Jagtap et al.^36^ used the Mini International Neuropsychiatric Interview (MINI) to highlight the most common perimenopausal complaints, which were found to be irritability (45%), headache (39.8%), body ache (34.3%), sleep disturbance (33.3%), and joint pains (35%). These physical aspects of perimenopausal depression are all considered and measured in the Meno-D.

The Meno-D will support a growing research field, interfacing both psychiatry and endocrinology, which indicates that perimenopausal depression is a unique subtype of depression requiring a different management approach, most likely with gonadal hormone treatment. Varied treatment options with mixed effects have been reported for perimenopausal depression symptoms. Selective serotonin reuptake inhibitors (SSRI) and serotonin noradrenaline reuptake inhibitors (SNRI) treatments remain the most popular pharmacological treatment choices for depressive symptoms during menopause, with varying outcomes^37^. However, hormone replacement therapy (HRT) has been demonstrated to improve or even replace SSRI treatment in women aged over 50 years^38,39^. A double-blind, randomized, placebo-controlled trial showed that transdermal estradiol treatment has significant antidepressant effect in depressed perimenopausal women^25^. Three case studies of women taking tibolone, an oral hormone treatment, describe improved mood within 6–8 weeks of taking tibolone^40^.

For women who prefer not to use medication to treat perimenopausal depression, a range of cognitive-behavioral, behavioral, and mindfulness-based therapies have been found effective in reducing severity of symptoms, especially cognitive-behavioral therapy (CBT)^37^. Of these non-pharmacological trials, CBT appears to have demonstrated the most beneficial effect and has been found to reduce depressive symptoms by at least 50% for half of participants, and achieve complete remission for just over 25% of participants^41^. Clinicians managing women in midlife, should consider perimenopausal depression to be a subtype of major depressive disorder and use a tailored treatment approach that takes note of menopause stage, psychosocial stressors, physical health, and past depression history.

There limitations in our study include the relatively small sample size and cross-sectional design. Using the Meno-D in larger sample sizes in the future would allow sensitivity and specificity analyses. Longitudinal studies would also allow for evaluation of the Meno-D sensitivity across STAW defined menopause stages, as well as for measurement of treatment response. Future research may also benefit from looking at the relationship between the Meno-D and lifestyle factors, such as alcohol and substance use.

In summary, the Meno-D provides a unique tool for clinicians, researchers, and women patients to measure the presence and severity of perimenopausal depression. Having a reliable tool to aid in the diagnosis of perimenopausal depression is very important in research of this relatively neglected area of women’s health. Clinically, it is very important to accurately detect and diagnose perimenopausal depression as early as possible to enable more specific treatments, such as hormone treatment strategies to be used. Early diagnosis of perimenopausal depression is critical to provide tailored treatments to improve the quality of life for women experiencing perimenopausal depression.

## Electronic supplementary material

Supplementary Material
