# Supplementary material for: Development and validation of a new rating scale for perimenopausal depression—the Meno-D
Source: Transl Psychiatry. 2018 Jun 28;8:123. doi: 10.1038/s41398-018-0172-0 (PMC6023883; doi:10.1038/s41398-018-0172-0)
Supplement: Supplementary file 1 — Supplementary Material [file 41398_2018_172_MOESM1_ESM.docx]

**Study 1: Clinical trials registration number ClinicalTrials.gov: *NCT01470092***

**Inclusion Criteria:**

- Females who are currently physically well and between 45 and 65 years of age
- Current DSM-IV diagnosis of depression disorder
- Able to give informed consent
- Perimenopausal as determined by symptom profile on the Stages of Reproductive Aging Workshop and gonadal hormonal profile

**Exclusion Criteria:**

- Known abnormalities in the hypothalamic-pituitary gonadal axis, thyroid dysfunction, central nervous system tumours, active or past history of a venous thromboembolic event, breast pathology, undiagnosed vaginal bleeding or abnormal Pap smear results in the previous 2 years.
- Patients with any significant unstable medical illness such as epilepsy and diabetes or known active cardiac, renal or liver disease; or the presence of illness causing immobilisation.
- Patients receiving treatment for depression including antidepressant medications, electroconvulsive therapy (ECT) / Transcranial Magnetic Stimulation (TMS), formal psychotherapy or counselling, within the past 6 months
- Patients experiencing severe melancholia, neurovegetative symptoms or current suicidality necessitating acute hospitalisation or intensive psychiatric treatment.
- Patients with psychotic symptoms or past history of severe mental illness including schizophrenia, and bipolar disorder.
- Use of any form of estrogen, progestin or androgen as hormonal therapy, or antiandrogen including tibolone or use of phytoestrogen supplements as powder or tablet
- Pregnancy / Lactation
- Smoking cigarettes and other nicotine products.
- illicit drug use and more than 3 standard drinks per day

**Study 2: Understanding Factors that Increase the Risk of Anxiety in Perimenopausal Women**

**Inclusion Criteria:**

- Females between 45 and 65 years of age
- Defined as peri menopausal according to the STRAW
- Able to give informed consent
- Females with Beck Anxiety Inventory (BAI) score ≤ 21 as the control group
- Females with BAI score >21 as the anxiety group

**3.6.2. Exclusion criteria:**

- Patients that are not perimenopausal according to the STRAW
- Patients that currently taking any form of estrogen, progestin and androgen as hormone replacement therapy
- Patient that currently taking anti-depressant medication
- Patients experiencing severe melancholia, neurovegetative symptoms or current suicidality necessitating acute hospitalisation or intensive psychiatric treatment.
- Patients that giving birth in past 1 month
